# Navigating the Shadows: A Comprehensive Review of Suicide in the Geriatric Population

**DOI:** 10.7759/cureus.53352

**Published:** 2024-01-31

**Authors:** Ateeba Ahmed, Pradeep S Patil

**Affiliations:** 1 Psychiatry, Jawaharlal Nehru Medical College, Wardha, IND

**Keywords:** age-friendly policies, social isolation, prevention strategies, suicide risk factors, elderly mental health, geriatric suicide

## Abstract

This review critically examines the complex landscape of suicide within the geriatric population, defined as individuals aged 65 and older. By synthesizing existing research, we elucidate critical findings related to the prevalence, risk factors, and challenges associated with suicide in this demographic. Social isolation, mental health issues, and the intricate psychosocial dimensions of ageing emerge as pivotal factors contributing to the vulnerability of older individuals. The conclusion underscores a compelling call to action, urging collaborative efforts from healthcare professionals, policymakers, and communities to implement targeted prevention strategies. Our vision for the future involves building a supportive and resilient community for the geriatric population, emphasizing age-friendly policies, robust social support networks, and destigmatizing mental health discussions. Through this comprehensive exploration, we aim to contribute to a deeper understanding of suicide in the geriatric population and inspire effective interventions that prioritize the well-being and dignity of older individuals.

## Introduction and background

The term "geriatric population" refers to individuals 65 years and older. This segment of the population is characterized by unique health challenges, including a higher prevalence of chronic illnesses, cognitive decline, and a variety of social and psychological factors that can impact their overall well-being [1]. Suicide among the geriatric population is a pressing public health concern that warrants careful attention. As societies worldwide witness an increase in life expectancy, the mental health and well-being of the elderly become paramount. Contrary to common misconceptions, suicide is not an inevitable consequence of ageing but rather a complex interplay of factors that can be identified and addressed [2].

The geriatric demographic faces a set of challenges that contribute to their vulnerability, such as social isolation, loneliness, physical health issues, and the loss of loved ones. Recognizing and understanding these challenges is crucial for developing effective preventive measures and support systems [3]. This comprehensive review aims to delve into the multifaceted aspects of suicide within the geriatric population. By synthesizing existing research, we intend to provide insights into the epidemiology, psychosocial factors, and prevention strategies related to suicide among older individuals. Our review will also explore the challenges in identifying and assessing suicide risk in this population, along with ethical considerations and future directions for research and policy.

## Review

Epidemiology of suicide in the geriatric population

Statistics and Prevalence Rates

The epidemiology of suicide within the geriatric population constitutes a significant public health concern. While global suicide rates have shown an overall decline, older individuals consistently exhibit the highest rates worldwide, with a discernible progression in suicide rates with advancing age, particularly among men [4]. In the United States, despite comprising only 12% of the population, older adults account for a disproportionately high 18% of all suicide deaths. The annual suicide rate for individuals over 65 surpasses 15 per 100,000, escalating with age to over 17 suicide deaths per 100,000 for those aged 75 to 84 [5]. In Italy, the statistics are equally troubling, with 1,775 deaths attributed to suicide after the age of 60 in 2013, representing 41.36% of the total, indicating a consistent rise in the elderly population over the past decade [6]. These figures underscore the alarming prevalence of suicide in the geriatric demographic, emphasizing the imperative for targeted interventions and enhanced mental health support within this population.

Risk Factors

Psychiatric disorders, particularly depression, pose a substantial risk for suicide among older individuals [7,8]. Understanding the nuanced connection between mental health and suicidal tendencies is crucial for the development of targeted interventions and support systems tailored to the unique needs of the geriatric population. In addition to mental health challenges, the presence of physical illnesses and somatic diseases significantly increases the risk of suicide, with this risk becoming more pronounced after the age of 85 [8,9]. Recognizing the intricate interplay between physical health and mental well-being is paramount for providing comprehensive and effective care for older individuals.

The experience of bereavement, specifically the loss of a spouse, emerges as a notable risk factor for suicidal death in the elderly [8,9]. Acknowledging and addressing the unique challenges faced by individuals coping with widowhood are crucial elements in suicide prevention efforts among older adults. Social isolation further compounds the risk of suicidal behaviour among older adults, especially those living alone [9]. Identifying and understanding the factors contributing to social isolation are essential for developing interventions that address the root causes and mitigate suicide risks within this demographic.

Moreover, access to lethal means, including firearms, presents a significant threat to the well-being of older individuals [9]. Tailoring preventive interventions to limit access to these means is essential in crafting effective suicide prevention strategies specifically designed for the geriatric population. It is important to underscore that interventions should be meticulously crafted to cater to the unique needs and challenges of the elderly, emphasizing targeted and nuanced approaches to reduce suicide risk [7]. Recognizing and comprehending these risk factors are pivotal steps in the development of precise prevention strategies aimed at enhancing the overall well-being of the elderly population.

Psychosocial factors contributing to suicide in the geriatric population

Social Isolation

Loss of social connections: The loss of social connections, resulting in social isolation, is a multifaceted challenge for older adults. This isolation can stem from various life events, including the death of a spouse, retirement, or physical limitations that impede their ability to engage in social activities [10,11]. The diminishing social network not only amplifies feelings of loneliness but also poses a substantial risk to mental well-being, potentially contributing to an increased vulnerability to suicide.

Loneliness: Loneliness is a pervasive experience among older adults, particularly poignant for those living alone or with limited social interactions. The emotional impact of loneliness extends beyond a mere absence of companionship, as it can foster deep feelings of hopelessness and significantly elevate the risk of suicide [10,11]. Addressing the root causes of loneliness becomes imperative in crafting interventions that aim to enhance the psychological resilience of older individuals.

Depression: Depression emerges as a formidable risk factor for suicide in the geriatric population, often triggered by a confluence of factors such as social isolation, cognitive decline, or other aging-related challenges [10,11]. Recognizing and addressing the complexities of depression in older adults is pivotal for formulating comprehensive preventive strategies that prioritize mental health and well-being.

Physical illness: The presence of physical illness, especially chronic or terminal conditions, introduces an additional layer of complexity to the emotional landscape of older adults. Such illnesses can contribute significantly to feelings of hopelessness and suicide risk, with the prevalence of somatic diseases in suicide cases escalating proportionally with age, notably spiking after the age of 85 [4]. A holistic approach to healthcare, considering both physical and mental aspects, becomes essential for addressing the nuanced needs of the elderly.

Financial issues: Financial concerns, encompassing limited income or the inability to afford necessary care, pose substantial stressors for older adults, potentially leading to heightened feelings of stress and hopelessness. This financial strain increases the risk of suicide among the elderly [10]. Recognizing the economic dimensions of mental health in older populations is crucial for implementing interventions that alleviate financial stressors and promote overall well-being.

Life changes: Significant life changes, such as the loss of a spouse or the inability to perform daily activities independently, can catalyze social isolation and, consequently, elevate the risk of suicide in older adults [10,11]. Acknowledging the profound impact of life transitions on mental health is pivotal for tailoring interventions that address the unique challenges faced by the elderly, fostering resilience, and mitigating suicide risk.

Loneliness

Loneliness stands as a noteworthy psychosocial element contributing to suicide within the geriatric population. Among older adults, social isolation and loneliness are prevalent concerns that can evoke feelings of hopelessness and despair. According to a narrative review, loneliness emerges as a distinct risk factor for suicide in this demographic [10]. The experience of loneliness can induce a sense of isolation and disconnection from others, further intensifying feelings of hopelessness and elevating the risk of suicide [10]. Notably, social factors, encompassing loneliness and social disconnectedness, serve as pivotal predictors of severe suicidal behaviors in older adults [12]. Loneliness intertwines with additional psychosocial risk factors, including depression, physical illness, and functional impairment [12]. Effectively addressing loneliness and social isolation among older adults becomes imperative for suicide prevention and the enhancement of overall mental health [10,11]. Psychosocial interventions, such as social support programs and counseling, play a crucial role in mitigating loneliness and other psychosocial risk factors in the geriatric population. Given that older adults may be more receptive to psychosocial treatments than pharmacological interventions, these strategies hold particular promise [11]. By strategically targeting loneliness and other psychosocial risk factors, it becomes feasible to diminish the risk of suicide in older adults and enhance their overall well-being.

Loss of Loved Ones

Psychosocial factors, such as the loss of loved ones, play a significant role in contributing to suicide within the geriatric population. Among the psychosocial risk factors associated with suicide in older adults are depression, physical illness, social isolation, and the inherent challenges of the aging process [4,10-13]. A comprehensive review of psychological autopsy investigations into senior suicides revealed a striking correlation, indicating that 71-95% of individuals who succumbed to suicide experienced severe psychological distress, with a predominant association between elderly suicide and the loss of a spouse [10]. The combination of social disconnectedness, somatic disease, and functional impairment emerges as a substantial risk factor for suicide [12]. Recognizing the limitations of medical treatment in older adults, there is a strong imperative to endorse psychosocial interventions as a viable approach [11]. In light of these findings, encouraging and implementing psychosocial interventions becomes paramount for addressing the multifaceted psychosocial risk factors associated with suicide in the geriatric population.

Mental Health Issues

Mental health issues: Mental health, particularly major depressive disorder, emerges as a profound risk factor for suicide among older adults. Notably, in psychological autopsy investigations scrutinizing senior suicides, a compelling correlation is evident, revealing that a striking 71-95% of individuals who died by suicide grappled with severe psychological disorders, predominantly major depressive disorder [10]. Recognizing the pivotal role of mental health, particularly depression, becomes imperative in devising targeted interventions aimed at mitigating suicide risk in the geriatric population.

Social isolation: Social isolation, characterized by social disconnectedness, a lack of social support, and pervasive feelings of loneliness, constitutes a significant contributor to the heightened risk of suicide in older adults. The impact of social factors on serious suicidal behaviors in this age group is underscored by empirical evidence [12]. Acknowledging and addressing social isolation are essential components in comprehensive suicide prevention strategies tailored for the unique needs of older individuals.

Physical illness: Physical health issues, encompassing chronic pain and disability, introduce an additional layer of complexity to the emotional landscape of older adults. Such physical ailments contribute to profound feelings of hopelessness and despair, thereby escalating the risk of suicide [11]. Recognizing the intersection of physical health and mental well-being is crucial in developing holistic interventions that address the multifaceted challenges faced by the geriatric population.

Life changes: Significant life changes, ranging from the death of a spouse and retirement to institutionalization, introduce profound emotional challenges for older adults. These life changes can evoke feelings of loss and despair, thereby elevating the risk of suicide [11]. Acknowledging the impact of life transitions on mental health is pivotal for crafting targeted interventions that address the unique emotional needs of the elderly, mitigating suicide risk, and fostering resilience in the face of significant life changes.

Financial issues: Financial struggles and economic challenges constitute significant contributors to heightened feelings of hopelessness and despair in older adults, thereby increasing the risk of suicide [10]. The strain of financial difficulties on mental well-being underscores the importance of addressing economic vulnerabilities in comprehensive suicide prevention strategies tailored for the geriatric population.

Alcohol abuse: Alcohol abuse emerges as a noteworthy risk factor for suicide in older adults, exacerbating feelings of depression and isolation [11]. The intersection of alcohol misuse with mental health issues underscores the need for targeted interventions that address substance abuse within the context of the unique challenges faced by older individuals.

Family discord: Family discord and conflicts within familial relationships contribute to heightened feelings of stress and despair in older adults, consequently elevating the risk of suicide [11]. Recognizing the impact of family dynamics on mental health becomes crucial in formulating interventions that address interpersonal conflicts and provide support to older individuals navigating familial challenges.

Cognitive deficits: Cognitive decline and dementia introduce another layer of complexity to the risk of suicide in older adults, as individuals may grapple with the challenges associated with changes in their mental and physical abilities [11]. A comprehensive approach to addressing suicide in the geriatric population necessitates tailored interventions that account for cognitive deficits, offering support, and fostering resilience in the face of these cognitive challenges.

Physical Health Challenges

Contributing psychosocial factors to suicide in the geriatric population encompass physical health challenges, psychiatric illness, recent loss, alcohol abuse, social isolation, perceived meaning of physical illness, family discord, retirement, cognitive deficits, and institutionalization. Physical health challenges, as established by empirical evidence [10-12], stand out as a notable risk factor for suicide among older individuals. Depression, a prevalent psychiatric illness in the geriatric population, poses a significant risk for suicide. Additionally, the combination of social disconnectedness with somatic disease and functional impairment emerges as a substantial risk factor for suicide [12]. Recognizing the limitations of medical treatment in older adults, there is a clear imperative to advocate for and implement psychosocial interventions [11]. Addressing these multifaceted psychosocial factors through tailored interventions becomes crucial for mitigating suicide risk and enhancing the overall well-being of the geriatric population.

Protective factors and resilience

Social Support Networks

Various studies have focused on exploring the protective factors and resilience exhibited by older adults, particularly within the framework of their social support networks. Research consistently demonstrates that protective elements, with a spotlight on social support, play a pivotal role in fostering resilience and lessening the impact of numerous crises on the mental health of elderly individuals [14]. Additionally, the correlation between psychological protective factors such as resilience, purpose-in-life, and social connections has been found to significantly influence caregiver mental health outcomes [15]. Moreover, a wealth of evidence indicates that a synergistic combination of protective factors, encompassing physical health, social support, and self-efficacy, contributes to the overall resilience of older adults [16].

Extending beyond individual factors, the cumulative impact of various psychosocial protective elements, prominently featuring social support, on selected health outcomes among older adults has been a subject of extensive study. These investigations underscore the substantial significance of these factors in promoting health and overall well-being in the elderly population [17]. Notably, the ongoing coronavirus disease 2019 (COVID-19) pandemic has brought to the forefront the critical need to recognize and fortify protective factors, particularly social support, among older adults. This proactive approach is crucial in mitigating the adverse impacts of social isolation and loneliness exacerbated by the pandemic [18]. The existing literature consistently reinforces the crucial role of social support as a fundamental protective factor. It not only promotes resilience but also acts as a mitigating force against the detrimental effects of diverse stressors on the mental health and overall well-being of older adults. These findings underscore the imperative of reinforcing social support networks and fortifying other protective factors to augment the resilience of the elderly population.

Access to Healthcare Services

Access to healthcare services is acknowledged as a crucial protective factor for older adults, representing a valuable resource that aids individuals in navigating challenging experiences and fostering overall health. Protective factors, defined as strengths or resources, play a pivotal role in helping individuals cope with adversities. Resilience stands out as a notable protective factor, presenting itself as a potential attribute to be cultivated in older individuals [19]. Resilience, rooted in adaptive coping mechanisms and positive attitudes, is recognized within models and nursing classifications that advocate for systematized care based on individualized needs, particularly benefiting vulnerable societal groups [16,19]. The confluence of protective factors, including enhanced emotional regulation, prosocial behavior, and resilience among older adults, serves as a catalyst for promoting health and facilitating successful aging [18,17,16]. The intertwining of these factors not only contributes to well-being but also aligns with models proposing systematic care tailored to individual needs, especially within vulnerable demographics in society.

Resilience Factors in Geriatric Individuals

The concepts of protective factors and resilience play a pivotal role in the promotion of mental health and overall well-being within the geriatric population. Protective factors, intrinsic attributes within individuals, contribute to reintegration and encompass self-reliance, self-efficacy, self-esteem, as well as psychological and physical health [16]. On the other hand, resilience represents the capacity to adapt and cope with adversity, shaped by both personal characteristics and environmental resources [16]. Together, these concepts become crucial tools for older adults in alleviating the effects of various crisis episodes and enhancing their mental health [14]. Examples of protective factors include purpose in life, resilience, optimism, an internal locus of control, and social connections [17]. By strengthening these effective protective factors, it becomes possible to enhance the resilience of elderly individuals, enabling them not only to weather difficult times but to thrive despite challenges [18]. Recognizing the significance of these concepts, particularly in the context of the geriatric population, underscores the importance of fostering a holistic approach that considers both individual attributes and external resources to support mental health and well-being in older adults.

Suicide methods and means in the geriatric population

Common Methods Used

The methods employed for suicide in the geriatric population exhibit variability, with certain patterns discerned through research. An investigation into the contemporary landscape of suicide methods among the elderly in England and Wales revealed that self-poisoning and hanging emerged as the most prevalent means of suicide in this demographic. Notably, there were notable distinctions in the suicide methods chosen by older men and women, as well as variations between older and younger individuals of both genders. Suicide by unspecified means was also frequent, particularly among older women [20]. Another study sought to delve deeper into the selection of suicide methods in individuals aged 65 years and above, aiming to provide additional insights into the specific approaches adopted by the geriatric population [21].

Crucially, it is important to highlight that the risk factors for suicide in the geriatric population encompass physical, familial, and financial challenges, in addition to feelings of hopelessness [10]. Gaining an understanding of the distinct methods employed for suicide within this population becomes essential for formulating targeted prevention strategies. Regrettably, for some cases, data on the potential method of suicide is unavailable, often labeled as "unspecified means." This underscores the critical need for accurate documentation of suicide methods to identify opportunities for prevention, emphasizing the importance of comprehensive and detailed reporting in suicide research [20].

Accessibility of Lethal Means

The accessibility of lethal means holds paramount importance in the landscape of suicide, particularly within the geriatric population. Lethal means encompass items or methods capable of causing death, such as firearms, medications, chemicals, and sharp objects [10]. Older adults, given their increased likelihood of having access to these means, are consequently more vulnerable to suicide. A study conducted by Amit et al. delved into the direct correlation between significant financial debt and suicidal tendencies in elderly individuals, elucidating that substantial financial burdens play a pivotal role in predicting both suicidal thoughts and actual suicide attempts [10].

An analysis of suicide trends among older individuals (65-79 years) and the oldest old (80 years and older) revealed hanging as the most prevalent suicide method for men, followed by self-poisoning, firearms, monoxide, and gas. For women, self-poisoning was the most frequently employed method [22]. This study underscored the imperative for a nuanced examination of the disparities in suicide trends between these age groups, aiming to delineate specific risk groups within this population particularly susceptible to suicide [22].

Yet another study sought to explore the selection of suicide methods in individuals aged 65 years and above [21]. The findings emphasized that comprehending the factors contributing to suicide in the geriatric population is imperative for healthcare providers, policymakers, and the broader community to take substantive action in preventing elder suicide [10]. Given the significant role of accessibility to lethal means, concerted efforts to prevent suicide among older adults must focus on identifying warning signs, understanding the factors influencing the choice of suicide methods, and implementing targeted interventions. These interventions should aim to reduce access to lethal means while providing appropriate support for older adults at risk [10,21,22].

Relationship Between Method and Gender/Age

The selection of suicide methods is significantly influenced by gender and age, delineating distinctive patterns in global suicide dynamics. On a global scale, men commit suicide at rates two to three times higher than women, with men often opting for more effective (lethal and fatal) methods such as firearms, hanging, and self-poisoning. Conversely, women tend to engage in more suicide attempts, frequently utilizing less lethal methods like self-poisoning [23]. A comprehensive study examining suicide methods among the elderly in England and Wales unveiled notable variations in the methods preferred by older men and women, as well as disparities between older and younger individuals of both genders. Hanging and self-poisoning emerged as the most prevalent methods for older people, while suicide by unspecified means was more common among older women than men [20].

Further research has underscored that male suicide attempts are consistently rated as more serious across various age groups and countries. Within each age category, males are more frequently categorized in the serious suicide attempt group compared to females [20]. These findings underscore the critical importance of factoring in gender and age disparities in the choice of suicide methods when formulating prevention strategies. Acknowledging these variations is imperative for developing targeted and effective approaches to prevent suicide, considering the unique dynamics associated with different demographic groups.

Challenges in identifying and assessing suicide risk in geriatric individuals

Stigma and Cultural Factors

Stigma and cultural factors wield considerable influence over the prevalence and manifestation of stigma related to mental illness and suicide. Within the Pacific Rim region, cultural elements like collectivism, Confucianism, face concern, familism, religion, and supernatural beliefs have been identified as contributors to stigmatizing behaviors and attitudes towards mental illness [24]. Likewise, in various cultures, negative stigma surrounding mental health symptoms or therapeutic services poses a significant barrier to seeking professional help. Research indicates that individuals from racial and ethnic minority groups are less inclined to seek outpatient therapy services due to cultural beliefs and the anticipation of discrimination [25].

Perceptions of suicide and mental illness can vary widely across different cultures. While some cultures may deem suicide an unforgivable sin, others may view it as a socially acceptable resolution to grief or the end of life [26]. These cultural variations can significantly impact help-seeking behaviors, the expression of distress, and the efficacy of suicide prevention and intervention efforts.

The stigma linked to help-seeking and mental illness, coupled with easy access to lethal means of suicide and inaccurate media portrayals of suicide, are identified by the CDC as cultural and environmental factors within broader societies that contribute to suicide risk [27]. Conversely, protective factors, including support from partners, friends, and family, a sense of connection to school and the community, and restricted access to lethal means of suicide, can help alleviate this risk [27]. Recognizing and addressing the influence of stigma and cultural factors on mental illness and suicide are imperative for developing effective, culture-specific interventions and fostering help-seeking behaviors within diverse communities.

Barriers to Communication

Communication about suicide in the geriatric population faces various barriers, encompassing refusal of help, challenges in offering assistance, language and cultural impediments, and the stigma associated with mental illness. These barriers pose significant challenges to the assessment and treatment of individuals at risk of suicide. For example, language and cultural barriers can create obstacles to meaningful communication essential for the effective assessment and management of suicide risk. Hence, utilizing qualified interpreters becomes crucial to ensure accurate and nuanced understanding [28]. Additionally, the stigma surrounding mental illness can act as a deterrent for individuals, preventing them from seeking treatment and disclosing their suicidal thoughts. This further complicates effective communication and intervention [29,30]. Recognizing and addressing these barriers is pivotal for enhancing suicide prevention and mental health care for the geriatric population.

Underreporting and Misdiagnosis

Identifying and assessing suicide risk in geriatric individuals presents significant challenges, marked by factors like underreporting and misdiagnosis. Research indicates that misdiagnosis, whether leaning towards over- or underdiagnosis, increases the risk of inappropriate or overlooked investigations and treatments. It also contributes to psychological distress and financial burdens for older patients [31]. Moreover, there are numerous instances of missed opportunities for suicide risk assessment in emergency and primary care settings. A recent meta-analysis revealed an overall pooled sensitivity of 41% in inquiries about suicidal thoughts [32]. Additionally, the detection of suicidality in older individuals faces obstacles such as covert symptoms going unrecognized and the absence of guidelines for suicide assessment by professional caregivers [30]. These challenges underscore the imperative for enhanced screening and assessment tools specifically tailored to meet the unique needs of the geriatric population.

Prevention strategies

Mental Health Awareness Campaigns

Campaigns focused on mental health awareness for the elderly population center around promoting emotional well-being, preventing suicide, and offering support for mental illnesses and substance use disorders. The World Health Organization (WHO) underscores the significance of strategies aimed at promoting mental health and preventing issues in older adults. These measures include initiatives to diminish social isolation, ensure secure and accessible housing, and provide adequate social support [33]. The WHO's Mental Health Gap Action Programme (mhGAP) incorporates interventions for psychosis, suicide, and substance use disorders in nonspecialized health settings, with a specific emphasis on the elderly [34].

Additionally, in the United States, the Substance Abuse and Mental Health Services Administration (SAMHSA) has orchestrated events like National Older Adults Mental Health Awareness Day. These events are designed to spotlight the unique needs of older adults and advocate for evidence-based approaches to suicide prevention, treatment, and recovery support [35,36]. These campaigns are crafted to address the distinctive mental health challenges encountered by the elderly population and offer resources to bolster their overall well-being.

Training for Healthcare Professionals

Training for healthcare professionals in geriatrics is imperative to meet the distinctive needs of older adults, and various initiatives and strategies are in place to enhance the education and skills of the healthcare workforce in this domain. Emphasizing the necessity to revamp the entire healthcare workforce in terms of geriatrics and gerontology is crucial to delivering specialized and quality care for older adults [37].

Efforts to fortify geriatric training for physicians are centered on bolstering the overall geriatrics medicine movement and creating specific incentives to encourage physicians to pursue careers in geriatric medicine [38]. Typically, geriatricians undergo a primary care residency followed by a geriatrics fellowship, where they gain insights into the conditions affecting older adults and the effects of aging on the human body and mind [38].

Initiatives prioritizing geriatrics in medical education and providing geriatric education for all clinicians, especially internal medicine and family medicine-trained physicians, play a pivotal role in enhancing care for older adults [39]. The American Academy of Family Physicians offers a Geriatric Medicine Self-Study program to assist family physicians in delivering optimal care to their elderly patients, covering various medical and mental health issues in the geriatric population [40]. These endeavors aim to equip healthcare professionals with the essential knowledge and skills to deliver high-quality care for older adults.

Access to Mental Health Services for Geriatric Individuals

Reducing financial insecurity and income inequality: Alleviating financial instability contributes to improved mental health among older adults [41]. Ensuring a stable financial foundation can positively impact various aspects of well-being, fostering a sense of security and reducing stress related to economic concerns.

Safe and accessible housing, public buildings, and transport: Establishing safe and accessible environments is crucial for promoting mental well-being among older adults [41]. Accessible living spaces and transportation options ensure that older individuals can navigate their surroundings with ease, fostering independence and minimizing potential stressors associated with unsafe or challenging environments.

Social support for older adults and their caregivers: Cultivating social connections plays a pivotal role in preventing loneliness and isolation, both of which can have detrimental effects on mental health [41]. Building a strong network of social support for older adults and their caregivers enhances emotional well-being and provides a crucial buffer against the negative impacts of social isolation.

Promoting healthy behaviors: Encouraging balanced diets, regular physical activity, and minimizing tobacco and alcohol use contributes significantly to overall mental health [41]. These health-promoting behaviors not only have physical health benefits but also positively influence mental well-being, creating a holistic approach to promoting a healthy lifestyle.

Mental health interventions: Employing a variety of mental health interventions, including mindfulness training, cognitive-behavioral therapy (CBT), well-being therapy, and social support, can be beneficial for older adults [42]. These interventions address various aspects of mental well-being, offering tailored approaches to support older individuals in maintaining and improving their mental health.

Resilience interventions: Programs dedicated to building resilience, such as mindfulness training, cognitive-behavioral therapy (CBT), and well-being therapy, are instrumental in assisting older adults in navigating mental health challenges [41]. These interventions focus on equipping individuals with coping mechanisms and strategies to enhance their resilience, fostering a positive approach to mental well-being.

Addressing dementia and abuse: Interventions aimed at preventing and responding to abuse, including mandatory reporting, self-help groups, helplines, and emergency shelters, play a vital role in safeguarding the mental health of older adults [33]. These initiatives create protective measures and support systems to address instances of abuse, promoting a safer and more secure environment for older individuals.

Access to quality mental health care: Ensuring that older adults have access to appropriate mental health care services is paramount for the maintenance of their mental well-being [33]. Providing accessible and quality mental health care services addresses the specific needs of older individuals, facilitating timely intervention and support.

Educating and raising awareness: Organizations like the Geriatric Mental Health Foundation actively work to dispel negative misperceptions surrounding mental health services and advocate for broader access to these services for older adults [42]. By promoting understanding and awareness, these initiatives contribute to reducing stigma and increasing the acceptance of mental health support within the elderly population.

Supporting caregivers: Offering resources and support for caregivers is essential to enhance their ability to support older adults with mental health needs [43]. Caregivers play a crucial role in the overall well-being of older individuals and providing them with the necessary tools and assistance ensures a more comprehensive approach to mental health care. Implementing these strategies allows communities and healthcare providers to collaborate in promoting mental health and preventing mental disorders in older adults.

Treatment and support for suicidal geriatric individuals

Psychotherapeutic Interventions

Adapted psychotherapy: Tailored to the specific needs of older adults, adapted psychotherapy focuses on addressing suicidal thoughts and underlying depression in this demographic. It involves a comprehensive exploration of the causes and therapy for suicidality in advanced age, taking into account factors such as method of choice and attitudes towards psychotherapy [44]. This personalized approach aims to provide targeted mental health support that aligns with the unique challenges faced by older individuals.

Psychosocial suicide prevention interventions: These interventions target various psychosocial factors contributing to suicidal thoughts in older adults, including anxiety, social isolation, loneliness, pain, disability, and institutionalization. Given the limitations of medical treatment in older adults, psychosocial interventions play a crucial role in addressing these factors and offering holistic support [11]. By addressing the broader context of mental health, these interventions aim to mitigate the risk of suicide in older adults.

Geriatric outreach programs: Geriatric outreach programs are designed to identify and support older adults at risk of suicide. These programs provide tailored interventions and resources, such as mental health assessments, medication management, and community-based care, to address the unique needs of older individuals [45]. By reaching out to this demographic, these programs contribute to early intervention and support.

Brief therapy: Studies indicate that brief therapy can be particularly beneficial for older adults, especially when combined with medication for depressive disorders [44]. This time-limited therapeutic approach focuses on addressing specific issues efficiently, making it well-suited for the older population, where time and resource considerations may play a crucial role.

Family involvement: Recognizing the significant role of family in the risk reduction and treatment of suicidal older adults, family involvement is a key component. Trained mental health practitioners, such as family therapists, can navigate issues related to the elderly, including depression and a high risk for suicide [45]. By involving the family, these interventions leverage existing support structures to enhance the overall well-being of older individuals.

Community-based care and mental health awareness: Adopting a comprehensive, multidisciplinary approach, community-based care and mental health awareness initiatives are crucial for addressing suicide in older adults [46]. This approach involves recognizing both physical and mental health problems in older adults, referring them for appropriate help, and training gatekeepers or para-professionals to identify and respond to suicide warning signs. By fostering awareness and community involvement, these initiatives aim to create a supportive environment for older adults, reducing the risk of suicide and improving their overall well-being.

Pharmacological Interventions

Pharmacological interventions play a crucial role in the treatment of suicidal geriatric individuals. Despite a substantial body of literature on population data, demographics, and suicide risk factors, there is a limited evidence base regarding effective pharmacological interventions for preventing suicidal behavior in older individuals [47]. Nonetheless, some studies have suggested the potential impact of certain pharmacological interventions, including antidepressants, antipsychotics, and other psychotropic medications, in reducing suicidal ideation and behavior among the elderly population [48,49].

It is imperative to exercise careful consideration when contemplating the use of pharmacological interventions, taking into account the individual's overall health, potential side effects, and the necessity for close monitoring, especially within the geriatric population. Additionally, assessing the effectiveness of these interventions should be done in conjunction with nonpharmacological approaches, such as psychotherapy and psychosocial interventions, to ensure comprehensive care for suicidal geriatric individuals [11].

Further research and clinical trials are essential to establish the efficacy and safety of pharmacological interventions specifically tailored to the needs of suicidal geriatric individuals. This research will contribute to the development of evidence-based guidelines for the use of pharmacological treatments in this vulnerable population [50].

Rehabilitation and Support Programs

Primary care-based programs: These initiatives focus on the early detection of suicidal ideation and the provision of appropriate interventions through primary care providers. Essential to these programs is the training of professionals to adeptly identify, intervene, and manage depression and suicide risk [51]. By integrating mental health awareness and intervention within primary care settings, these programs aim to facilitate timely support for older adults.

Community programs: Community-based workshops and outreach initiatives play a vital role in reducing the social stigma associated with mental health issues and enhancing access to support services for older adults [10]. These programs contribute to building a supportive community environment that fosters mental well-being and encourages individuals to seek assistance without fear of judgment.

Group activities: Involving older adults in group activities is an effective strategy to enhance social connections, diminish isolation, and alleviate symptoms of depression [45]. By creating opportunities for social engagement, these activities promote a sense of belonging and camaraderie among older individuals, contributing to their overall mental health.

Telephone counselling: Providing accessible mental health support through telephone counseling services offers older adults a convenient avenue for seeking assistance and accessing valuable resources [45]. This mode of counseling addresses potential barriers to in-person services, ensuring that older individuals have readily available support when needed.

Physical activity: Encouraging physical activity has demonstrated efficacy in preventing suicidal ideation among older adults [47]. Regular exercise not only contributes to physical well-being but also positively impacts mental health, making it a valuable component in comprehensive suicide prevention efforts targeted at the geriatric population.

Collaborative management: Emphasizing collaborative management involves close coordination with mental health professionals, including geriatric psychiatrists, to ensure holistic care for older adults facing suicide risk [10]. This approach recognizes the importance of a multidisciplinary effort in addressing the complex factors contributing to suicidal ideation and behavior in the geriatric population.

Geriatric-specific programs: Tailored to the distinctive needs of older adults, these programs comprehensively address factors such as physical, mental, and social losses, along with tackling social isolation and loneliness [10]. By specifically targeting the unique challenges faced by the elderly, these programs aim to provide more effective and relevant support for this demographic.

Gatekeeper training: Training professionals, particularly primary care providers, in detecting and managing depression and suicide risk among older adults plays a crucial role in enhancing access to care and reducing the risk of suicide [51]. Ongoing research and evaluation are essential to determine the effectiveness of these programs in preventing and managing suicidal ideation and behavior in older adults. Additionally, addressing barriers to program access and identifying specific beneficial components can further enhance the impact of rehabilitation and support programs [45].

Family and Caregiver Involvement

Empowerment approach: Advocating for an empowerment approach involves actively engaging family caregivers in suicide prevention efforts, leading to improved outcomes and enhanced support for the at-risk individual [52]. This approach entails understanding the caregiver's perspective and equipping them with the necessary resources and training to effectively support the person in need [53].

Suicide risk assessment: Family members and caregivers serve as invaluable sources of collateral information for assessing suicide risk, given their close involvement in the individual's life and ability to recognize warning signs [54]. Their insights can contribute significantly to a comprehensive understanding of the risk factors involved.

Strength-based strategies: Involving family and caregivers in interventions harnesses their strengths and resources, such as emotional support, encouragement, and practical assistance, contributing to the prevention of suicide [54]. Leveraging these inherent strengths can positively impact the well-being of the at-risk individual.

Training and education: Providing family members and caregivers with training and education on suicide prevention enables them to identify warning signs, deliver appropriate interventions, and navigate the complexities of the mental health system [51]. This knowledge empowers them to play an active role in the prevention process.

Support groups and resources: Offering access to support groups, resources, and professional assistance for family members and caregivers aids in managing their own emotions and needs, ultimately enhancing their ability to support the individual at risk [54]. By incorporating family and caregiver involvement, particularly in the geriatric population, into suicide prevention strategies, we empower these key stakeholders with the essential resources, training, and support needed to identify, prevent, and manage suicide risk in older adults.

## Conclusions

In conclusion, this comprehensive review illuminates the intricate landscape surrounding suicide within the geriatric population. The synthesis of research has highlighted the multifaceted nature of this issue, emphasizing the crucial role played by social isolation, mental health challenges, and the unique psychosocial dynamics of ageing. A resounding call to action reverberates throughout this discourse as we confront these sobering realities. Healthcare professionals, policymakers, and communities must unite in a concerted effort to address the pressing issue of suicide among older individuals. This call extends beyond raising awareness to implementing tangible prevention strategies, encompassing community-based interventions, targeted mental health campaigns, and improved access to services tailored to the specific needs of the elderly. However, amidst the challenges lies a beacon of hope. The future holds promise as we aspire to build a supportive and resilient community for older individuals. By enacting age-friendly policies, strengthening social support networks, and destigmatizing mental health discussions, we can envision a future where the geriatric population thrives, and the specter of suicide diminishes. In this vision, the agency and dignity of older individuals are paramount, guiding us towards a society that acknowledges their experiences and challenges and actively engages in solutions, fostering an environment where they not only survive but flourish.
